# Investigating the predictive power of seismic statistical features using ensemble learning

**DOI:** 10.1371/journal.pone.0342765

**Published:** 2026-02-19

**Authors:** Wei Quan, Denise Gorse

**Affiliations:** Department of Computer Science, University College London, London, United Kingdom; Ministry of Education, MOROCCO

## Abstract

Earthquake prediction is an extremely challenging problem, one that has been in the past (and sometimes still is) claimed to be impossible. Given this undisputed high level of difficulty, work that reports a high level of prediction success might reasonably be regarded with a degree of caution. We will discuss here how these results may in many cases be due to data leakage. However, a recent paper co-authored by one of us has shown a promising level of predictive ability even when its methodology strictly controls for possible overfitting and data leakage. We here build on that prior work by asking if the demonstrated predictive value of the seismic statistical features used there is due to their being able to capture domain-specific knowledge. Specifically, we compare the value of the same set of 60 seismic statistical features used in the aforementioned previous work to the value of a set of 428 generic time series features from the tsfresh package. We train an XGBoost model to predict if there will be an earthquake of magnitude *M* ≥ 5 in the following 15 days, and find models using the seismic statistical features can attain AUCs of up to 0.87, while models using the tsfresh features alone cannot obtain results substantially better than random. It therefore does appear that seismic-specific catalogue features are able to capture valuable information about subsurface conditions prior to an impending earthquake. We do not attempt to carry out operational earthquake prediction, considering it premature at this time. However, the demonstrated seismic-specific origin of the predictive power of our features gives hope that by augmenting and enhancing them such prediction may become feasible, and we conclude by discussing some novel directions for future work.

## Introduction

Earthquake prediction, ideally specifying when and where the predicted future event will occur, with what magnitude, and with what probability [1], is clearly a highly desirable goal, but one that is also highly challenging. Indeed, it was claimed in 1997 that making predictions with sufficient reliability to be of any practical use was ‘effectively impossible’ [2]. However, in the new era of big data, many problems that were intractable in the past, such as protein folding, as described in [3], have yielded to advanced machine learning models. There has recently been a rapid expansion in available seismological data [4,5], and this has given hope of similar advances in earthquake prediction [6]. There has been an exponential growth over the last 10 years in works devoted to this topic [7], though given the difficulty of the problem, and the inevitable ethical and legal issues should any machine learning (ML) model be actively deployed, it would seem best to proceed cautiously, using interpretable ML models where possible and using strict methodological controls in regard to overfitting and data leakage. In this regard, we note that test results in some earthquake prediction papers are very high, with accuracies in excess of 95% sometimes reported, and there is strong evidence of data leakage in some cases. Additionally, Zhao and Gorse [8] showed explicitly that handling test data correctly (with the test period in the future of the data used for training the model) makes a large difference to the quality of the results. Training on the past and predicting the future is a basic for time series problems yet this principle is surprisingly often not adhered to in works focused on earthquake prediction.

However, [8] also showed that even when strict methodological controls are in place, seismically-focused statistical features derived from earthquake catalogue data do appear to have predictive value. We here build on that work by asking where this predictive value comes from. We compare the predictive ability of the 60 seismic statistical features used in [8], originating in 2018 in [9] and since used in a number of other works, with those from a package that offers hundreds of generic time series features. We will show that while these generic features have very little ability to predict earthquakes, the seismic statistical features appear to have a degree of ability to do so, even when using the same strict controls as in [8]. It thus appears that changes in sets of seismically-focused features can be used to measure a build-up of subsurface stresses, and hence that it may be possible, with future work, to use changes in these ‘seismic statistical features’ (SSFs) as effective predictors of future seismic events.

Specifically, in this paper:

- We ask if the predictive value evidenced in [8] of the 60 SSFs presented first in [9] leverages seismological insight. We evidence this is so by comparing the value of these 60 features with that of 428 generic time series statistical features offered by the tsfresh [10] package, which latter are shown near-wholly ineffective.
- We investigate the ranking of seismic features by our models, gaining a degree of insight into the mechanisms by which these features are being used for prediction.
- We make suggestions for future work, focusing on moving from regional prediction (as here) to more localised prediction, additionally incorporating spatiotemporal features which, when combined with a number of other proposed improvements, could feasibly progress toward actionable predictions.

The remainder of this paper will be structured as follows. Background and related work will give necessary background and review some related work. Methodology will explain our methods, including data sources and train/validation/test partitioning, while Results will present and analyse the results of our experiments. The following Discussion section will discuss these results, additionally looking at limitations of the current prediction framework and how these could be overcome in future work, before we finish with our Conclusion.

## Background and related work

### Introduction to seismic statistical features

Seismic statistical features (SSFs), sometimes also known as ‘seismic indicators’, take on many forms, from very simple, such as the largest earthquake experienced in the region of interest over the last seven days, to much more complex measures. The best-known SSF is the ‘*b*-value’, which derives from the Gutenberg-Richter (GR) rule [11],

$$log_{10}N(M)=a-bM,$$ (1)

where *N*(*M*) is the number of earthquakes with a magnitude greater than or equal to *M*, and *a* and *b* are constants. The *b*-value has been studied extensively over many years, recently, for example, in [12] [13]. *b*, which averages globally to a value of around 1.0, is widely considered to be an (inverse) measure of crustal stress. When *b* is large, larger events have been proportionally more frequent, releasing stress, while when *b* is small, the converse is true, and hence stress is building. This relationship between the value of *b* and stress has been evidenced both in natural [14] and laboratory [15] systems. There has been a recent, effective proposal, the Foreshock Traffic Light System (FTLS) of Gulia and Wiemer [16] [17], to use changes in *b* as a means to decide when a large event can be safely classed as the main shock (increase in *b* of ≥10%), as opposed to being only a foreshock of an even larger event (decrease in *b* of ≥10%). However, outside of the context of the FTLS work (determining the likelihood of an imminent larger event associated with the same fault), there has been debate about the value of *b* for earthquake prediction; for example [18] and [19] are equivocal about the value of *b* and [20] and [12] are substantially sceptical. There has been equivalent, though less extensive, discussion in the case of the *z*-value, a measure of seismic rate change introduced by Habermann [21]. This feature measures the difference between two means, a recent mean defined for a shorter period that might precede a seismic event, and an earlier mean, defined over a longer period, used to determine a background seismicity, and will be positive when the seismicity rate decreases, negative when it increases. Oynakov and Botev [22] found that larger seismic events in the southern Balkans tended to occur in areas of relatively high *z* and relatively low *b*-value, but this observation has not been more widely supported, while Bodri et al. [23] pointed out that studies of the predictive significance of the *z*-value in the Aegean area may have been influenced by human factors, including events that resulted in the closure of seismic monitoring stations.

It is highly likely that any successful prediction of seismic events in a more general context would need to be based not on changes in single SSFs, or on the behaviour of small sets of such features, such as *b* and *z*, but on subtle changes across many features, interacting in a complex way. Thus, any machine learning approach should seek to incorporate as many relevant seismic statistical features as possible. As well as adding the Gutenberg-Richter *a*-value and *β*, an alternative measure of seismic rate change introduced by Matthews and Reasenberg [24], we might consider many other seismic catalogue features, ranging from simple (for example, *x*_6_, the magnitude of the largest event experienced in the last seven days, introduced by Reyes et al. in [25] to allow the prediction model to potentially learn the essence of the Omori-Utsu [26] and Bath’s [27] laws) to more complex (for example, the probabilistic recurrence times of Wiemer and Wyss [28]). All of these are considered in the current work. We might also consider features derived from the location and depth information in earthquake catalogues, such as those suggested in [29], but this is out of the scope of the current work. (Though will be a key topic addressed in the future work part of the Discussion.)

### Related work

The problem of earthquake prediction from SSFs is usually framed as ‘will there be an earthquake in the region of interest of magnitude ≥M during the next *D* days?’, based on a set of features computed from the last *N* catalogue events. The number of SSFs considered in this type of ML-based earthquake prediction work has ranged from the small numbers used in earlier work (the six parameters used by Ma et al. in 1999 [30], expanded to eight by Panakkat and Adeli in 2007 [31]; the seven parameters proposed by the Reyes group in 2013 [25]), to much larger sets such as the 60 parameter set first introduced in 2018 by Asim et al. in [9], defined in detail in [32], and also used in this current work. While the Asim set is one of the largest, there may now be a move toward even larger datasets that include non-catalogue information alongside catalogue-derived features, such as the recent paper of Hu et al. [33], which combines catalogue features with spatial (geological and seismic) data.

As well as a use of larger and more diverse feature sets, there has also been a trend toward more complex prediction models. These have the potential to be of great value but require a degree of caution. Datasets of the order of hundreds or thousands of examples, as have been typically used by models making earthquake predictions from SSFs, are not ‘big data’, and care needs to be taken when using deep learning models to avoid overfitting. It is also important with any type of model to avoid data leakage. The most common way this occurs in SSF-based earthquake prediction relates to the use of random test sets, after first creating a set of examples by calculating features based on a moving past-time window. As this window advances, one event at a time, most of the information in consecutive feature vectors will be the same. This in turn means that if a random subset of the examples is assigned to be the test set, many of these will have much in common with examples used for training, severely impairing the interpretation of the test results. This phenomenon was demonstrated explicitly by Zhao and Gorse in [8], which examined the difference in test performance when a random 30% test set was converted to a temporal one (final 30% in time), when some instances of moderately strong prediction, enabled by data leakage from train to test set, were reduced to ones close to guesswork when this data leakage was prevented. This problem is widespread in the field. Of the 33 pre-2024 papers reviewed in [8], 7/33 either stated their test data selection was non-temporal, or this was discovered to be so after downloading available code, while a further 7/33 left the origin of their test data unclear. Hence, approaching half of the published works surveyed in [8] had at least potential data leakage and it will be seen the situation (summarised in Table 1) is not dissimilar in the case of the six 2024–25 papers considered below.

**Table 1:** Summary of results from 2024-25 earthquake prediction papers that use seismic statistical features. Authors are listed alphabetically. ‘Split type’ refers to the splitting of the dataset into (train, validation [if used], test) and is one of {temporal, random, unknown}. ‘Model’ is the one for which the paper reported the best results. ‘Target 1’ is the condition under which the model output should be 1/‘yes’, as framed for the closest-given magnitude threshold to our *M* = 5. ‘Test performance low–high’ gives the lowest and highest test performance values reported (potentially depending on the time horizon *D* and geographical region). Abbreviations: DL = deep learning; ANN = artificial neural network.

Authors	Ref.	Split type	Model	Target 1	Test performance low–high
Hu et al.	[33]	temporal	multi-modal DL	≥5	accuracy 80.42%
Mukherjee et al.	[34]	unknown	XGBoost	≥5	accuracy 91.89%–94.38%
Ommi et al.	[35]	random	ANN	≥5	accuracy 93.02%
Peng et al.	[36]	unknown	V-detector-bagging	≥4.5	AUC 0.75–0.81, accuracy 77.6%–79.2%
Yavas et al.	[37]	random	Random Forest	4.72–5.50	accuracy 98.20%
Zhao & Gorse	[8]	temporal	CatBoost	≥5	AUC 0.51–0.88, accuracy 61.9%–82.7%

To begin with those works that use temporal splitting, Hu et al. [33] explicitly states the calendar periods corresponding to train, validation, and test, which makes the nature of the split very clear. It could be noted that there are no gaps between these data subsets, which potentially could lead to some data leakage. However, a lookback period of up to a year is used in the computation of certain features and so gapping would necessarily have involved discarding a substantial amount of data. The model proposed in [33], SafeNet, is a multi-model deep learning (DL) model that fuses catalogue-derived features capturing information over short, medium, and long timescales with features extracted from geological and seismic maps, the resulting set of 282 features being the largest SSF-containing feature set of which we are aware. The paper is of additional interest in that it divides its target area, China, into 85 4^°^ × 4^°^ seismically active regions and makes predictions separately for each of them. It is clearly of greater value to make more localised predictions but it is inevitably also more challenging than the whole-region predictions that are the norm in SSF-based prediction studies. The paper makes predictions of four classes (0≤M<5, 5≤M<6, 6≤M<7, M≥7) but also gives an average accuracy of 80.29% for a magnitude threshold of 5, which is the value quoted in Table 1.

Unlike [33], Zhao and Gorse [8], which shares its 60 seismic features with [32] and related works from this group, does not subdivide its three geographical regions of interest (Chile, Southern California, and Hindukush) into smaller regions. However, it uses gapping (of 50 events, the window size used to calculate seismic features) between its train, validation, and test sets, and also, as mentioned previously, demonstrates explicitly the effect of data leakage due to a random selection of test set. As this paper will be the foundation for the work later presented here, and will be referred to frequently in various necessary contexts, we will not discuss it further here, beyond noting that results were heavily dependent on the quantity and quality (in terms of magnitude of completeness) of the available data, with the lowest performance for with the Hindukush region (4,351 examples, *M*_*c*_ = 4.0) and the highest performance for Southern California (33,544 examples, *M*_*c*_ = 2.6).

Moving now to those two papers in Table 1 that do not make the nature of the data split clear, the first of these, Mukherjee et al. [34], has a number of commonalities with [8] and with this current study. It is a whole-region (the Himalayan seismic belt and its surroundings) study that uses a near-identical feature set to ours, aside from its omission of *β*, an alternative measure of seismic rate change introduced by Matthews and Reasenberg in [24], and insertion of *μ* (the mean time, during the past event window, between earthquakes of a magnitude considered typical for that region) and *c* (a measure of deviation during this period, for these typical events, from an ideal recurrence model). *μ* and *c* were introduced by Panakkat and Adeli in 2007 [31], the other six in the eight-feature Panakkat and Adeli set in fact dating back to Ma et al. in 1999 [30]. A number of models were trialled in [34], but the best-performing was XGBoost, the model we will also use here. However, it is not clear how the 25% test data in [34] were selected, and test accuracies for the four magnitude thresholds (4, 4.5, 5, 5.5) and four prediction horizons (7, 10, 15, and 30 days) are high, no less than 91.89% (for a 10-day prediction horizon) in the case of a magnitude threshold of 5. We also note that cross-validation was performed for the 75% train set in order to select model hyperparameters, with up to 12 folds being used. The value of this depends on how the data were partitioned. If the dataset were shuffled, with a random 25% removed for testing, the validation portions in the folds are likely to overlap significantly with the training portions and cross-validation might in this case even increase overfitting. If the data were partitioned in a temporal manner, while this is a correct separation of the test data it means also that cross-validation needs to be performed in a manner designed for time series data, with gapping between training and validation portions and with no folds in which a part of the training data is in the future of the validation data. As with other aspects of the data handling, it is not clear if this was done.

Also within this group of works for which the mode of data splitting is unclear, the work of Peng et al. [36] is of interest for its use of a model unusual in earthquake prediction, V-detector-bagging, which is a form of artificial immune system model augmented by bagging. This paper uses the smallest feature set of those studies listed in Table 1, only the eight Panakkat and Adeli [31] indicators, predicting, for two regions of China (Sichuan and Xinjiang), if an event of magnitude ≥5 will occur within the next month. The relatively modest results for the 30% test sets, compared with those reported in some other works reviewed here, suggest temporal splitting may have been used, as do snapshots of temporally ordered data in Tables 6 and 9 of [36]. But as in the case of [34] it is not stated explicitly that this is so.

Ommi et al. [35] is an instance of a work that does not state the test data are randomly chosen, but for which examination of the downloadable code shows this to be so. Prediction is on the basis of a small feature set, the eight Panakkat and Adeli features [31], plus also *dE*^1/2^ [38], a measure of seismic energy release. The work makes one-month-ahead predictions, based on conditions within the previous month, for the north Zagros region using three different predictive models, of which a small multilayer neural network (two hidden layers of 32 neurons) performed best. Most experiments are done with a lookback window of 100 events and a prediction threshold of 5.5, but there is a test result quoted for *M* = 5 (our choice of magnitude threshold) using a lookback of 150 events, which is the result quoted in Table 1. Test results are averages over five test folds, with 20% of the data allocated for testing in each fold. The result for *M* = 5 of 93.02% appears excellent, aside from caveats related to those raised for [34] (unless it is clearly stated the splitting method is one designed for time series, even if temporal ordering were retained, 4/5 of the folds would involve training at least in part on the future while testing on the past). However, given that the code indicates the test sets are chosen randomly, the result is unfortunately undermined by an issue of data leakage.

The single paper listed in Table 1 that clearly states that the selection of the (20%) test data is random is that of Yavas et al. [37]. This work uses 21 features, including members of the Panakkat and Adeli [31] and Reyes [25] sets, together with other catalogue-derived features such as the rolling mean of earthquake depth over the last 30 days, as input to a Random Forest model, to predict seismic activity in the Los Angeles region of southern California for the following 30 days, assigning future events to one of six magnitude classes (0.65–2.82, 2.82–3.25, 3.25–3.67, 3.67–4.13, 4.13–4.72, 4.72–5.50). We will consider Class 6 (4.72–5.50) to be closest to the problem as framed here, which asks if there will be a future event of magnitude ≥5 in the following 15 days. The Class 6 problem in [37] may be considered easier than ours because the prediction time horizon is longer. However, it also has both an upper and a lower bound (as do all the classes in [37]), which makes it considerably harder. The six-class-averaged test accuracy reported in [37] was 97.69%, with an accuracy of 98.20% for Class 6 (112/115 samples assigned correctly). Overall the results reported in this work are exceptionally high, in line with their having benefitted from data leakage.

Reflecting on Table 1 and the associated discussion, in which in 2/6 cases it was impossible to know for certain how the test data were chosen, we would urge researchers in this area who have, in fact, split their data in the correct temporal way to make this very clear, lest readers consider the omission of this information, in combination with promising results, to be a warning flag. A further methodological issue in the above-reviewed studies pertains to the catalogue magnitude of completeness *M*_*c*_. This is the magnitude below which a magnitude-frequency plot does not follow the Gutenberg-Richter distribution, below which not all events will have been recorded due to instrumental limitations. The Gutenberg-Richter parameters *a* and *b* (and hence also any other feature that depends on them) by their nature cannot be accurately estimated from data that do not obey the Gutenberg-Richter law. For this reason catalogue data for magnitudes lower than an estimated *M*_*c*_ should be discarded before calculation of *a*- or *b*-dependent seismic statistical features. Of the six papers listed above, only [8] states that it carries out this recommended truncation of the original catalogue data; [33,36], and [35] do not mention the topic; and [34] and [37] make it conversely clear they use all of the available data. For models with a large and diverse feature set, with many features that do not depend at all on *a* and *b* (such as that used in [33]), the inclusion of events with *M*<*M*_*c*_ is likely to be a minor source of noise. However, for models with fewer, SSF-dominated features, the introduced noise may be more damaging, and estimation of *M*_*c*_ and exclusion of events with *M*<*M*_*c*_ (as is done, for example, in [32] and in other works from that group) is in any case best practice for models that use the Gutenberg-Richter *a*- and *b* values in any way.

To conclude this section on related work, as mentioned earlier the 2021 paper of Al Banna et al. [39], though previously discussed in [8], is of interest to review it here because it, too, uses generic statistical features as well as SSFs. The objective in [39] is to use features computed from a 50-event sliding window to predict if there will or will not be an earthquake in the Bangladesh region in the following month, using an attentional deep learning (DL) model, with a separate DL model used to predict the distance of any such event from Dhaka, the capital city and a major population centre. The paper uses a small number of SSFs, the eight-feature Panakkat and Adeli set [31], alongside a small number (2–20, depending on the method of feature selection) of features from the HCTSA package [40], a source of generic time series statistical features. The eight-feature SSF set used in [39] is considerably smaller than the 60-feature set used here (discussed in Seismic statistical features (SSFs)), as is the set of generic features (we use 428 as input to our models). The sources of the non-SSFs also differ, as we use the tsfresh package [10], discussed in tsfresh statistical features (TSFs), rather than HCTSA. The work of [39] further differs from ours in the way features are selected. In the case of the eight Panakkat and Adeli SSFs there is no feature selection. In the case of the HCTSA features, the original 7,700 features are reduced to 2–20 before input to the models. In contrast, in our work here (as described in Feature selection) we use the model itself to help decide which features, from both the 60-feature SSF and 428-feature tsfresh sets, are important, allowing for the possibility of exploiting non-linear dependencies among them. However, despite the differences in model and methodology between the work of [39] and ours, there is a notable commonality in that Al Banna et al. also found less value in non-seismic features when compared, in their case, to the value of the eight-feature Panakkat and Adeli set.

## Methodology

**Fig 1:**
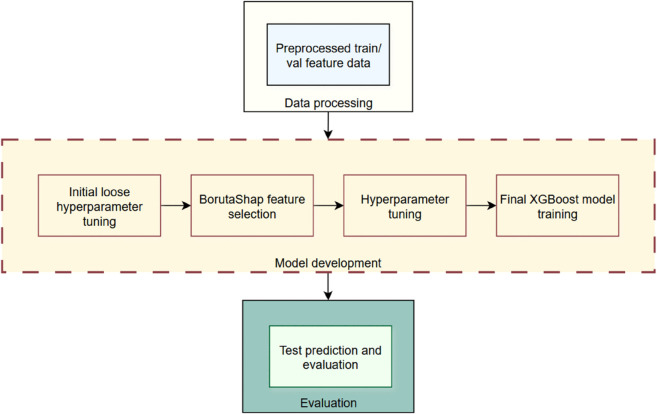
Complete modelling pipeline.

As in [8], we frame the problem as one of short-term prediction based on features extracted from the recent seismic record. Specifically, we ask whether or not there will be a seismic event of magnitude M≥5 in the next 15 days. We compare the predictive value of SSFs to that of generic time series statistical features from the tsfresh [10] package. For each region considered (Japan and Chile), we will make predictions on the basis of seismic statistical features alone, seismic features plus all (428) applicable features from the tsfresh package, and tsfresh features alone. As well as considering overall success in terms of ROC curves and AUC values, we will look at usage of features, including, in the mixed-input scenario, the proportions of features selected by the model from the SSF and tsfresh sets. Our overall modelling pipeline is shown in Fig 1. All analyses were conducted in Python 3.8 using an Intel i7 processor and NVIDIA RTX 4060 GPU.

### Data

**Fig 2:**
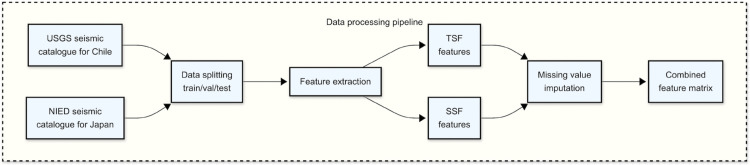
Data processing pipeline.

This subsection will look at data sources, data partitioning (into train, validation, and test), and the statistical features (both SSFs and tsfresh features) that were calculated from the raw catalogue data. This data processing pipeline is shown schematically in Fig 2. It should additionally be noted that scaling (standardisation) was carried out for the SSF feature data, but using the mean and standard deviation from the training set when processing the validation set, and mean and standard deviation from the entire training/validation set when processing the test set, in order to avoid data leakage.

#### Data sources.

**Fig 3:**
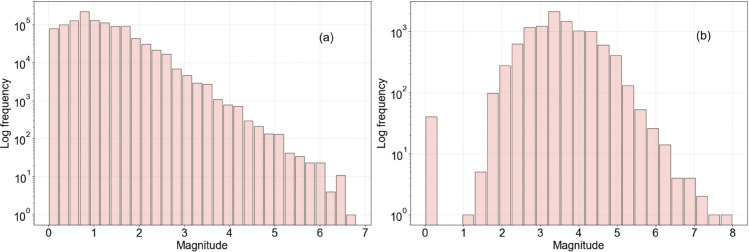
Magnitude-frequency histograms for the studied regions. (a) Japan; (b) Chile.

Japan and Chile were chosen as the regions of study due both to the high levels of seismicity in these areas and the quantity and quality of available catalogue data. Japan in particular has a very extensive network of sensitive seismic detectors, and so the dataset from this region is both large and inclusive of many smaller events that would not be detected elsewhere. In the case of Japan, data were acquired from the Japanese National Research Institute for Earth Science and Disaster Resilience (NIED); Chilean data were acquired from the U.S. Geological Survey (USGS). Magnitude-frequency histograms for these datasets are provided in Fig 3(a) (Japan) and 3(b) (Chile). The magnitude of completeness *M*_*c*_ was estimated for Japan to be 1.0 and for Chile to be 3.4. Summary information about the dataset before splitting into training, validation, test is given in Table 2. It can be seen that for Japan especially there is more than sufficient data to train an ensemble model like XGBoost; we would not expect overfitting to be a risk (though we in any case check for this via a validation dataset).

**Table 2:** Summary information about the two earthquake catalogue datasets used in this study.

Region	Source	Coordinates	Period	Number of examples
Japan	NEID	30.1^°^ N, 45.9^°^ N,128.6^°^ E, 146.0^°^ E	01/10/2000-31/12/2012	559,787
Chile	USGS	32.8^°^ S, 36.1^°^ S, 70.4^°^ W, 72.8^°^ W	06/01/1980-18/05/2024	5,924

#### Data partitioning.

For each of our datasets, we split data on a temporal basis, as in [8], in order to avoid data leakage. Before feature generation, the catalogue is split temporally into training/validation (70%) and test (30%) sets. To avoid data leakage, a gap of 50 events (the window size used in the seismic feature computations) separates train/val and test. The train/val set is further split into training and validation sets on the same 70%-30% basis, with again a gap of 50 events. The resulting partitions are given in Table 3.

**Table 3:** Partitioning of the two earthquake catalogue datasets.

Region	Train	Validation	Test
Japan	274,211	117,540	167,936
Chile	2,818	1,229	1,777

### Features used

Before separately discussing our use of seismic and generic statistical features, it should be noted that one question occasionally raised in earthquake forecasting work with catalogue-derived features is whether the data should be declustered (have foreshocks and aftershocks removed, leaving only the mainshock in each seismic sequence). While most papers in the field do not discuss this issue, some do, and conclude that declustering should be done (for example [41]). However, many of the SSFs depend specifically on a measurement of precursory activity (e.g., *T*, the time in days over which the last *N* events occurred, and *x*_6_, the largest magnitude in the last seven days). Additionally, Mukherjee et al. [34], who do discuss the issue, provide sound arguments for the retention of aftershocks, citing Gitis et al. [42] (who argued the removal of aftershocks decreased prediction quality via the reduction of available data) and Taroni & Akinci [43] (who concluded that removing aftershocks underestimated seismic hazard). We would ourselves add to these arguments that the labels (foreshock, mainshock, aftershock) can anyhow only be attached to events post facto, such that removing data on this basis—requiring one to effectively look into the future—might also be deemed a form of data leakage. Hence, while we truncate the catalogue data to remove events for which *M*<*M*_*c*_, we do not here decluster.

#### Seismic statistical features (SSFs).

There are many possible seismic features that can be calculated from earthquake catalogue data, even in the purely temporal domain. However, because of the number of past studies that have used them, we in this work chose to use the 60 seismic statistical features first introduced in [9]. A full definition of all these 60 features will not be given here as these features are described in detail in [32]. However, it should be noted that any feature that incorporates the Gutenberg-Richter *a*- and/or *b*-value, referred to in [32] as a ‘parametric’ feature, is represented twice in the feature set, depending on whether *a* and *b* were calculated using the method of maximum likelihood estimation (MLE) or of least squares (LSQ). Since there were 27 (from an original 33) such features, the total rises from 33 to 60 (6 non-parametric features + 2 × 27 parametric features). We note that within the seismological community MLE is strongly favoured over LSQ as a means of calculating *a* and *b*. However, we chose to follow the route first established in [9] and present features calculated using both methods, on the basis that a model with feature selection should be able to decide which were the most useful features to retain. This decision is supported by the cross-correlation analysis of features in Mukherjee et al., [34], which as noted in Related work, uses a near-identical feature set to ours, including the dual-mode calculation of parametric features. This analysis shows that while correlations between MLE and LSQ variants of features are substantial (e.g., 0.81 for *a*, 0.79 for *b*, 0.62 for *M*_*def*_, 0.53 for *x*_7_), they are not so large that could confidently say it was possible to discard one of the two variants as informationally redundant. Matlab code [44] was provided by the authors of [32] in order to calculate their 60 features. This code has been used here, aside from two features (*z* and *β*) for which the code revealed data leakage in that the background seismic rate was calculated using the entire available dataset including the test set, and hence used data from the future of the current time point. *z* and *β* were therefore recalculated from the raw catalogue data using Python to use information only from the past in training and validation. The full set of 60 seismic features we use, divided into parametric (Table 4) and non-parametric (Table 5) features, are listed below, where ‘feature’ here refers to the way features will later be referred to in Fig 5. (We deviate from the nomenclature of [32] in that we name *a* and *b* as parametric; this seems logical as while these depend only trivially on the Gutenberg-Richter intercept and slope parameters, it is unarguable that they do so.)

**Fig 4:**
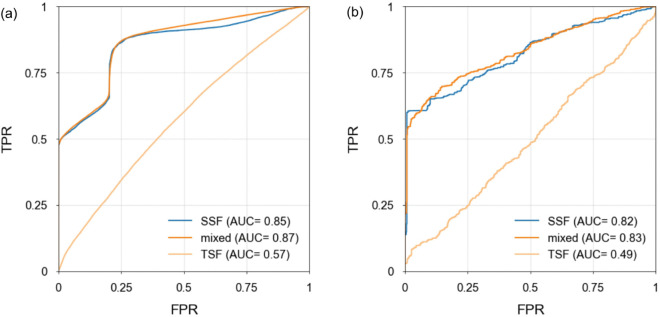
Test period ROC curves and AUC values. Results are shown for (a) Japan and (b) Chile. In each case three input scenarios are considered (seismic statistical features (SSFs) only, SSFs + generic tsfresh statistical features (TSFs), TSFs only), predicting if there will be an event of magnitude ≥5.0 in next 15 days.

**Fig 5:**
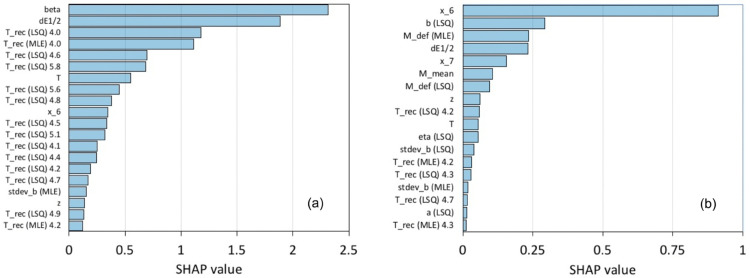
Top-ranked test period SSF-only feature importances. Results are shown for (a) Japan (top 20 of the 39/60 SSF features selected during validation) and (b) Chile (all 18/60 SSF features selected during validation), predicting if there will be an event of magnitude ≥5.0 in next 15 days.

**Table 4:** Parametric (*do* depend on the Gutenberg-Richter *a*- and *b*-values) seismic statistical features (SSFs), here calculated by both the maximum likelihood (MLE) and least squares (LSQ) methods.

Feature	Description	Origin
a, b	Gutenberg-Richter (G-R) *a*- and *b*-values	[45]
stdev_b	standard deviation $\sigma_{b}$ of the *b*-value	[46]
T_rec M′	probabilistic recurrence time $T_{rec}=\frac{T}{10^{a-bM^{\prime }}}$, for $M^{\prime }$ in {4.0, 4.1, 4.2, ... , 6.0}	[28]
eta	deviation *η* from G-R law during last *N* events	[30]
M_def	magnitude deficit $M_{def}=M_{max,actual}-M_{max,expected}$	[30]
x_7	probability *x*_7_ according to the G-R law of an earthquake with magnitude M≥6.0	[25]

**Table 5:** Non-parametric (*do not* depend on the Gutenberg-Richter *a*- and *b*-values) seismic statistical features (SSFs).

Feature	Description	Origin
z	seismic rate change $z=\frac{R_{1}-R_{2}}{\sqrt{\frac{S_{1}}{n_{1}}+\frac{S_{2}}{n_{2}}}}$ (method of Habermann)	[21]
beta	seismic rate change $\beta =\frac{M(t,\delta )-n_{tot}\delta }{\sqrt{n_{tot}\delta (1-\delta )}}$ (method of Matthews & Reasenberg)	[24]
dE1/2	seismic energy release $dE^{\frac{1}{2}}=\frac{\sum \left(10^{\left(11.8+1.5M\right)}\right){\;}^{\frac{1}{2}}}{T}$	[38]
T	time *T* (in days) during which last *N* events occurred	[30]
M_mean	mean magnitude *M*_*mean*_ of last *N* events	[30]
x_6	largest magnitude in last 7 days, *x*_6_	[25]

#### tsfresh statistical features (TSFs).

The Python package tsfresh [10] is a source of generic time series features, drawn from many areas of time series analysis. The tsfresh package was used here in preference to the HCTSA package [40] used in [39] because its being written in Python allowed it to be more easily integrated into our pipeline. We note also a use of tsfresh in [47] to compute features potentially predictive of future volcanic eruptions, though the package is in this case applied to raw seismic data, not catalogue data, and the application area, while still within seismology, is somewhat different. The tsfresh package contains around 750 different features, but some of these require data points that are evenly spaced in time and so not applicable to seismic catalogue data. Removing the inapplicable features left 428 generic statistical features that might potentially be useful for the prediction of a seismic event. These tsfresh features were computed with a rolling window of *N* = 50 events, the same as in the case of the seismic statistical feature computation. We note that the inbuilt ‘impute’ function in tsfresh was rewritten so as to be based only on past data, in line with our intention to be rigorous in excluding potential sources of data leakage, a past problem in a substantial number of machine learning studies in this area, as noted in Related work.

### Machine learning model

While there has been a huge increase in the amount of seismological data available for training ML models [4,5], in most regions of the world there is not yet sufficient data to support attempts at earthquake prediction from raw catalogue data using deep learning (DL) models. Models with inputs based on extracted features are at the moment more practical, and for prediction only from small numbers (order of 10s) of such pre-calculated features we would argue that DL models, at the moment infrequently used in this field, are unlikely to be helpful beyond increasing the risk of overfitting. Tree-based ensembles, in contrast, have had wide usage in this area and have shown their effectiveness for prediction from seismic time series features in both natural (e.g. [48,49,50,8]) and laboratory [51] settings, and were the choice of 3/6 of the recent SSF-based prediction papers reviewed in Table 1. Here we use XGBoost [52], on the basis that it is a popular model of this class that is sufficient for our core purpose of showing that catalogue-derived seismic features have excess value above that of generic time series features. We do not exclude the possibility of future work with DL models. However, it seems to us the true value of deep learning is when data are not only numerous but rich (the multimodel DL paper of Hu et al. [33], with its access also to image data, being an example of this), and hence will reserve DL models for future work that goes beyond seismic catalogue features alone.

### Feature selection

As noted in the description of Features used, there were a total of 488 available features (60 from the dataset of [9], 428 from tsfresh). In all three experimental scenarios (seismic features only, seismic features + tsfresh features, tsfresh features only) we used Boruta-Shap feature selection [53] during training and validation to prune back the number of input features and gain insight into the basis for model decisions. (It should be emphasised that the feature importances in Fig 5 reflect only the usage of features during the test period, for each of the two studied regions; there is no feature selection performed with respect to the test data.)

### Prediction time horizon and magnitude threshold

The most common framework for prediction of seismic events from earthquake catalogue data is to ask ‘will there be an event of magnitude ≥M in the next *D* days?’ The most frequently used time horizon is 15 days, used for example in [32] and [8]; this frequency of use is the reason we adopted *D* = 15, also. In relation to the choice of threshold magnitude *M*, we did not want to unnecessarily complicate the investigation by introducing issues generated by class imbalance. We therefore chose a value of *M* = 5.0, as this would for both the Chile and Japan regions give a more or less equal chance there would, or would not, be an earthquake ≥M in the next 15 days.

### Hyperparameter tuning

The selection of appropriate hyperparameters (parameters that affect the outputs of the model but which cannot be learned during model training) is a critical process in machine learning model development, as these govern the behaviour of the underlying model with respect to performance, training efficiency, and model generalisation to unseen datasets. The selection procedure employed in this study comprised two stages of hyperparameter optimisation: preliminary ‘loose’ tuning and fine tuning. Prior to feature selection, the ‘max_depth’, ‘n_estimators’, and ‘learning_rate’ hyperparameters of XGBoost were loosely tuned to ensure correct model behaviour. This initial optimisation was performed to conserve computational resources, given the presence of hundreds of features in both the tsfresh and mixed feature scenarios. The same loose tuning procedure was applied to the SSF scenario to maintain methodological consistency, even though there were significantly fewer features in this case. Following this initial optimisation, features were selected using the BorutaShap algorithm, on the basis of validation set performance, before the fine tuning process was conducted. The individual hyperparameters, their search ranges, and the optimal values identified for each region are given in Tables 6 and 7.

**Table 6:** Hyperparameters, search ranges, and optimal values identified during the fine tuning process for Japan (*M* = 5.0).

Hyperparameter	SSF		tsfresh		SSF + tsfresh	
	Range	Optimal	Range	Optimal	Range	Optimal
n_estimator	{1500...2000}	1700	{1000...1800}	1300	{1200...2000}	1600
max_depth	{2, 3, ..., 10}	2	{2, 3, ..., 10}	2	{2, 3, ..., 10}	2
learning_rate	{0.1, 0.01}	0.1	{0.1, 0.01}	0.01	{0.1, 0.01}	0.1
min_child_weight	{1...8}	8	{1...8}	8	{1...8}	3
gamma	{0...8}	8	{0...8}	5	{0...8}	8
subsample	{0.5...1}	0.7	{0.5...1}	0.5	{0.5...1}	0.5

**Table 7:** Hyperparameters, search ranges, and optimal values identified during the fine tuning process for Chile (*M* = 5.0).

Hyperparameter	SSF		tsfresh		SSF + tsfresh	
	Range	Optimal	Range	Optimal	Range	Optimal
n_estimator	{100...800}	410	{10...500}	20	{10...500}	20
max_depth	{2, 3, ..., 10}	2	{2, 3, ..., 10}	2	{2, 3, ..., 10}	2
learning_rate	{0.1, 0.01}	0.01	{0.1, 0.01}	0.1	{0.1, 0.01}	0.1
min_child_weight	{1...8}	6	{1...8}	3	{1...8}	8
gamma	{0...8}	0	{0...8}	0	{0...8}	5
subsample	{0.5...1}	0.5	{0.5...1}	1	{0.5...1}	0.5

## Results

### Model performance

Fig 4 shows test period ROC curves for each category of features (60 seismic features only, 60 seismic + 428 generic tsfresh features, 428 tsfresh features only), for each of our two studied regions. It is immediately apparent that the models using the tsfresh features (TSFs) alone fail at usefully predicting future seismic events, since for neither region studied does the AUC value exceed 0.57, while models using the seismic features (SSFs) alone have AUCs in excess of 0.82 for both Japan and Chile. In the mixed-features scenario, when the models are able to pick freely from either the seismic or the generic time series feature sets, the AUCs for both regions are very similar to those obtained using seismic features alone. The very small positive differences (0.02 for Japan, 0.01 for Chile) are, to our mind, an insufficient justification for the inclusion of additional features that have no clear seismological interpretation, nor do they seem to capture orthogonal variance, either.

One might also notice that both the hyperparameter-optimised tsfresh-only and mixed-features (SSF + tsfresh) models have low capacity (n_estimators = 20, max_depth = 2) in the case of Chile, which contrasts with the model configuration in the case of Japan (for tsfresh-only, n_estimators = 1300, max_depth = 2; for SSF + tsfresh n_estimators = 1600, max_depth = 2). We attribute this notable difference to a combination of a very large difference in dataset size (5,924 examples for Chile in comparison to 559,787 for Japan) and the use of features with only weak predictive value (which in the SSF + tsfresh case primarily act as noise). A model with a relatively small dataset that is additionally given weakly predictive features to learn from is highly prone to overfitting. Because of this, the hyperparameter optimisation process selects a simpler model than might otherwise be expected. We note that a smaller n_estimators search range was used in the case of Chile, even in the SSF-only case. We carried out preliminary experimentation with larger ensemble estimators, but it became quickly evident that the small size of the Chile dataset could not support a model as complex as that which proved optimal for Japan; for this reason the n_estimators search range, for the detailed phase of hyperparameter optimisation, was for Chile restricted on the grounds of computational efficiency to one within which the optimal configuration would be likely to be found.

Our use of generic time series features was motivated by their success in domains such as financial forecasting, where some of these features effectively capture latent non-linearities in complex signals. We noted also that tsfresh had been successfully used in an application in volcano monitoring [47]. However, our results highlight a potentially critical theoretical distinction regarding signal characteristics. Financial data—and in fact the data used in [47] also—typically represents a dense state evolution, while in contrast earthquake catalogues are inherently sparse, marked point processes. The generic feature extraction algorithms (e.g., spectral analysis, autocorrelation) used by tsfresh are theoretically capable of capturing complex non-linear dynamics without physical assumptions, and we hypothesised they might adapt to irregular sampling through appropriate binning. However, our results suggest these methods struggle to extract meaningful patterns when the underlying data structure consists of sparse, discrete events rather than continuous signals. When applied to spatiotemporally sparse seismic bins, these algorithms likely ‘dilute’ the discrete, bursty signal into statistical noise. We therefore believe that, unlike SSFs, which are designed to aggregate discrete events according to specific scaling laws (e.g., Gutenberg-Richter, Omori), generic time series features likely fail to preserve the sequential and magnitude-dependent information necessary to identify precursory stress accumulation.

While we do not favour the use of accuracy as a performance metric for even slightly imbalanced data (at a magnitude threshold of *M* = 5 the IR for the Chilean dataset is around 2:1, while for Japan the classes are roughly balanced), it is interesting to compare the test accuracies obtained here with those listed in Table 1. For the SSF-only model with which we are going forward, the test accuracies were 85.5% for Japan and 72.2% for Chile, values notably in line with the lower values quoted for the two papers for which a temporal data split is certain (Hu et al. [33] and Zhao & Gorse [8]).

### Feature importances

As mentioned in Feature selection, not all of the available features were used in any of the three scenarios; a validation set was used during the training phase to select, on the basis of SHAP value, those features found most useful. Notably, in the mixed-features scenario, proportionally far more of the seismic features than the generic features were selected: for Japan, 37/60 (62%) of the SSFs were picked, as opposed to 149/428 (35%) of the TSFs; for Chile, 15/60 (25%) of the SSFs were picked, as opposed to 63/428 (15%) of the TSFs. Looking at the top 20 features used during the test period in this mixed-features scenario was additionally instructive; only two of the top 20 features were TSFs in the case of Japan, indicating a strong preference for the model to make use of the seismic features. This further evidences that it is unlikely to be helpful to include generic time series features alongside seismic ones, especially when desiring an interpretable earthquake prediction model.

Given the above-mentioned desire for interpretability, it is of interest to see which of the SSFs are found most valuable for prediction, and the test period feature importances of Fig 5(a) and 5(b) address this, listing for Japan (a) the 20 top-ranked SSF-only features, and for Chile (b) all 18 of the SSF-only feature selected during validation. (As previously emphasised, test period feature importances look only at the test period usage of features; feature selection is done purely on the basis of validation data.)

It is striking that for Japan (Fig 5(a)) there are many uses of the recurrence times of Wiemer and Wyss [28], first used as input to an ML earthquake prediction model in 2018 in [9]. However, recurrence times are not greatly prominent in the case of Chile (Fig 5(b)), with only five of the 18 features selected by the model for this region being of this type. Aside from the difference in completeness of the catalogue (*M*_*c*_ estimated as 1.0 for Japan, 3.4 for Chile), it seems possible, given the remarks in [28] about differences in frequency-magnitude distribution even over a few kilometres, that the difference between the regions is due to the much higher density of seismological stations in Japan, reported in 2020 in [54] to have 700 permanent stations with an average inter-station distance of around 20 km. In contrast there are around 300 real-time permanent stations in the case of Chile [55], with only temporary networks, such as that which monitored the Atacama Fault System between March 2010 and March 2012, having inter-station distances in the order of tens of kilometres [56]. Better data quality (in terms of both geographical coverage and magnitude of completeness) may allow a more effective use of these potentially valuable seismicity indicators.

Data quality differences between the Japanese and Chilean datasets might also explain why the seismicity rate feature *β* [24] is ranked top in Japan but is not selected during validation in the case of Chile, while conversely *x*_6_, the largest magnitude during the last seven days, is top for Chile but not ranked very highly for Japan. The computation of *β*, which uses Poisson/binomial variance in order to calculate a background rate of seismicity, is vulnerable to noise in smaller datasets, and the dataset for Chile is only around 1% of the size of the dataset available for Japan. In contrast, *x*_6_ is a very simple measure of precursory seismicity that would be expected to be robust even for smaller datasets such as the one used here for Chile. In this context, we note that *x*_6_ was also found to be the most important feature for Chile in both [8] (which used the same 60 SSFs as in this paper) and [57] (which had a substantially different set of features but included *x*_6_ among them). We also note that *x*_6_ was top-ranked in the paper of Mukherjee at al. [34], which made predictions for the Himalayan seismic belt using a dataset of 14,994 examples, much closer to the size of our smaller USGS Chilean dataset than our Japanese NEID one.

As discussed in Features used, all features including at least one of the Gutenberg-Richter parameters *a* and *b* were, following [9], calculated in two different ways, the method of least squares (LSQ) and the method of maximum likelihood estimation (MLE), in spite of the fact that the latter is preferred within seismology. This allowed the models to choose which variations were more effective, which from a machine learning point of view is a sound approach, as prior beliefs about the values of features can be wrong. However, we did not expect to see such a marked preference for the LSQ mode of computation. This was especially true for Japan, where 13/16 (81%) of the parametric features in Fig 5(a) used LSQ, rather than MLE, in contrast with 8/11 (73%) for Chile. While MLE is a more accurate method, it is also more sensitive to non-stationarity than LSQ, which could be a larger concern for a dataset limited in time (the Japanese dataset covered only 12 years) than in the case of Chile, where the catalogue covered 44 years. We would note also that there is a difference between assessing the value of a feature in isolation and assessing that same feature’s usage by a complex model that simultaneously also considers many other features. In future work we in any case plan to move away from either the LSQ or MLE computation of the Gutenberg-Richter *a* and *b* parameters to the use of their now-preferred replacements a-positive [58] and b-positive [59], which are more suited to rolling-window computation within earthquake catalogue data.

It might finally be noticed that the Gutenberg-Richter slope parameter *b* is not among the top 20 feature importances for Japan (Fig 5(a)). This initially surprised us, given the evidence that changes in *b* can contain valuable precursory information, and that the Japanese dataset is of especially high quality in terms of size and completeness. However, those situations in which changes in *b* have been observed to be an effective predictor, as in [16] and [17], are very different from the whole-region predictions attempted here and in the many previous works that use a similar framework. In our future work, discussed in the section to follow, we aim to move away from whole-region prediction, which is used here only for investigating the predictive value of SSFs, and would be disadvantageous to attempts to predict future seismic activity in an operational forecasting task. We aim to move to a more local approach that incorporates spatial and temporal elements in the construction of features. We would then expect to find *b* ranked highly in all geographical regions.

## Discussion

This study set out to investigate the comparative value of large sets of both domain- specific seismic statistical features (SSFs) and generic statistical features from the tsfresh [10] package (TSFs), for the task of earthquake prediction based on seismic catalogue time series data. From the results we have presented, based on 100,000s of test examples in the case of Japan, it does appear that seismic statistical features may be able to capture valuable information relevant to future seismicity while generic time series features cannot. This was not an obvious conclusion, as no previous study has allowed SSFs and TSFs competitive access to the data, as in our ‘mixed’ scenario, with the machine learning model itself able to choose which features, or combinations of features, were most valuable to use. However, while it therefore indeed appears SSFs are able to exploit some proxy knowledge about subsurface processes preparatory to a seismic event, there are limitations to this study, some are common to the framework from which it was derived, which we aim to address in future work:

- Catalogue completeness (the value of *M*_*c*_) fluctuates over time for a number of reasons (e.g., changing sensitivity of instrumentation), but our current means of calculating the parameters *a* and *b* assumes it to be stable.
- The event window size *N* is here assumed to be 50, in common with the many past works reviewed in [8], but this value is unlikely to be ideal for modern, more complete catalogues such as the Japanese NEID one.
- We did not remove aftershocks in the test set during evaluation, in order to avoid data leakage in this proof-of-concept framework that prioritises the investigation of the predictive values of SSFs and TSFs on all general seismicity. In future work that moves toward operational earthquake forecasting sub-analyses removing immediate aftershock windows will be needed, to distinguish predictions of new stress build-ups from Omori law decay.
- Past events used to calculate the seismic features are not filtered with respect to distance; currently even events highly unlikely to be relevant on the grounds of distance make contributions to the feature calculations.
- Only temporal information is used to calculate features, when studies such as [29] indicate that spatial information, such as the volume of the 3D convex hull of relevant hypocentres, is also very important.

In relation to the first of the above limitations, as remarked in the Results section, we intend to follow the trend in computational seismology and replace the Gutenberg-Richter *a* and *b* parameters by the derived quantities a-positive [58] and b-positive [59], which are more suited to rolling window computation and require only a rough estimate of *M*_*c*_. In relation to the second limitation, the optimal size of the past event window *N* will in future be adjusted on the basis of validation set AUC, as from a machine learning point of view it is clear it should be treated as a model hyperparameter. We believe, however, that the final two limitations are the most significant and pressing to overcome. In future work we intend to move away from whole-region prediction and are developing a locally-focused framework that includes relevant spatiotemporal features and respects plausible spatial distance constraints. This new methodology will very likely result in a much stronger representation of some familiar seismic features, in particular the Gutenberg-Richter *b*-value, which in itself, under certain circumstances, as in the recent work of Gulia and Wiemer [16] [17], has been able to make successful predictions of future activity. Even so, we would expect the true power of the developed machine learning model to be in its ability to exploit nonlinear combinations of both familiar and novel features, which in the latter case could include, for example, the fractal dimension of rock fracture networks [60].

## Conclusion

We have shown that a relatively simple and interpretable machine learning model, XGBoost, when given a selection of earthquake catalogue time series features, shows a persuasively strong preference for features with a seismological, rather than generic time series statistical, basis, and that generic features do not add complementary information to domain features. Moreover, we have shown that even though the modelling framework used here is the less-favourable whole-region one historically typical of works that attempt to predict earthquakes from seismologically-derived features, it is possible to make 15-day forecasts with a meaningful, if not yet actionable, level of success. On this basis, we believe there is a hope that moving to a more localised spatiotemporal framework (with appropriately chosen additional spatial features) will result in substantial improvement, even if the long-sought goal of effective operational earthquake forecasting may remain out of reach for considerably longer yet.

## Availability of code

The code used to generate the results of this study may be downloaded from: https://github.com/erinuclkwon/ssf_tsf.
